# Role of Nrf2 in Lipopolysaccharide-Induced Acute Kidney Injury: Protection by Human Umbilical Cord Blood Mononuclear Cells

**DOI:** 10.1155/2020/6123459

**Published:** 2020-07-28

**Authors:** Li-Xin Feng, Fei Zhao, Qian Liu, Jin-Cheng Peng, Xiang-Jie Duan, Ping Yan, Xi Wu, Hong-Shen Wang, Yin-Hao Deng, Shao-Bin Duan

**Affiliations:** Department of Nephrology, The Second Xiangya Hospital, Central South University; Hunan Key Laboratory of Kidney Disease and Blood Purification, Changsha, Hunan 410011, China

## Abstract

**Background:**

Acute kidney injury (AKI) is one of the common complications of sepsis. Heretofore, there is no effective treatment for septic AKI. Recent studies have revealed that besides treating hematological malignancies, human umbilical cord blood mononuclear cells (hUCBMNCs) show good therapeutic effects on other diseases. But whether hUCBMNCs can protect against septic AKI and its underlying mechanism are unknown.

**Methods:**

The rat model of lipopolysaccharide- (LPS-) induced AKI was developed, and the injection of hUCBMNCs was executed to prevent and treat AKI. ML385, a specific nuclear factor E2-related factor 2 (Nrf2) inhibitor, was used to silence Nrf2. The cell experiments were conducted to elaborate the protective mechanism of Nrf2 pathway.

**Results:**

An effective model of LPS-induced AKI was established. Compared to the rats only with LPS injection, the levels of inflammation, reactive oxygen species (ROS), and apoptosis in renal tissues after hUCBMNC injection were markedly attenuated. Pathological examination also indicated significant remission of renal tissue injury in the LPS+MNCs group, compared to rats in the LPS group. Transmission electron microscopy (TEM) showed that the damage of the mitochondria in the LPS+MNCs group was lighter than that in the LPS group. Noteworthily, the renal Nrf2/HO-1 pathway was activated and autophagy was enhanced after hUCBMNC injection. ML385 could partly reverse the renoprotective effect of hUCBMNCs, which could demonstrate that Nrf2 participated in the protection of hUCBMNCs. Cell experiments showed that increasing the expression level of Nrf2 could alleviate LPS-induced cell injury by increasing the autophagy level and decreasing the injury of the mitochondria in HK-2 cells.

**Conclusion:**

All results suggest that hUCBMNCs can protect against LPS-induced AKI via the Nrf2 pathway. Activating Nrf2 can upregulate autophagy to protect LPS-induced cell injury.

## 1. Introduction

Sepsis is a life-threatening organ dysfunction caused by a dysregulated host response to infection [1]. Acute kidney injury (AKI) usually occurs in patients suffering from severe sepsis. AKI is a common complication of sepsis for critically ill patients, with an incidence up to 50%, and the mortality rate of patients with AKI and sepsis is significantly higher than that of patients with AKI alone [2, 3]. Hence, more effective strategies are needed for the treatment of AKI after sepsis.

At present, the relevant mechanism of AKI after sepsis has not been fully elucidated, which may involve inflammation, oxidative stress, microcirculation dysfunction, apoptosis, autophagy, and other aspects [4]. As an important molecule that mediates several oxidative stress pathways, the contribution of nuclear factor E2-related factor 2 (Nrf2) is of particular interest. Several studies have shown that autophagy is significantly decreased in AKI after sepsis [5–7]. Nevertheless, whether the Nrf2 pathway can protect LPS-induced AKI via autophagy is uncertain.

Human umbilical cord blood mononuclear cells (hUCBMNCs) are mononuclear cells derived from the cord blood, which comprise multiple cells, including immature immune cells, hematopoietic stem cells (HSCs), mesenchymal stem cells (MSCs), and endothelial progenitor cells (EPCs) [8]. Previous studies have depicted that hUCBMNCs have significant beneficial effects on relieving damages in middle cerebral artery occlusion, erectile dysfunction, diabetic nephropathy, and ventricular function in rat models [9–12] and improving prognosis in chronic complete spinal cord injury patients [13], whereas no relevant studies show whether stem cells can alleviate septic AKI.

Therefore, we made a hypothesis that hUCBMNCs could protect against LPS-induced AKI by regulating the Nrf2 pathway. To test this hypothesis, AKI rat and cell injury models were built using LPS. Besides, we determined whether hUCBMNCs could protect against LPS-induced AKI by modulating the Nrf2 pathway and compared the ineffective effects of hUCBMNCs with ML385, a specific Nrf2 inhibitor. Furthermore, cell experiments were conducted to elaborate the protective mechanism of the Nrf2 pathway.

## 2. Materials and Methods

### 2.1. Animals and Drugs

All animal experiments were conducted following the guidelines on animal care of the Second Xiangya Hospital of Central South University. Adult 8-week-old male Sprague Dawley rats, weighing approximately 240–260 g, were purchased from Hunan Slac Jing Da Laboratory Animal Co., Ltd. (Changsha, China). The animal ethics committee of Hunan Slac Jing Da Laboratory animal Co., Ltd. approved the experiments (IACUC-SJA18055). All rats in the experiments were given free access to water and normal rat chow and acclimatized for seven days before each animal experiment. Rats were sacrificed by 300 mg/kg chloral hydrate. Subcutaneous injection of 5 mg/kg carprofen was executed before the chloral hydrate used. AKI is defined as an increase in SCr ≥ 50% from the baseline value within 48 hours in the Kidney Disease Improving Global Outcomes (KDIGO) [14]. ML385 (Selleckchem, Houston, TX, USA; Cat# S8790) was dissolved in DMSO. LPS (Escherichia coli, serotype 0111: B4; L2630, Sigma-Aldrich) was dissolved in normal saline (NS).

### 2.2. Separation and Extraction of hUCBMNCs

The hUCBMNCs were provided by Guangzhou Cedicine Bio Tech Co., Ltd. As described previously, human umbilical cord blood (hUCB) was collected by a 250 ml standard blood collection bag (Baxter, Deerfield, IL) which contains a citrate-phosphate-dextrose anticoagulant [15]. Pathogens of communicable diseases were tested after the collection, which include hepatitis B and C, cytomegalovirus, human immunodeficiency virus, and Treponema pallidum. The umbilical cord blood was processed within 24 hours after collection and then the mononuclear cells (MNCs) from the umbilical cord blood were separated by Ficoll-Paque TM PLUS (Amersham Bioscience, Uppsala, Sweden). According to the flow cytometry result, the hUCBMNCs mainly exhibited CD38, CD5, and CD3 positive and CD34- and CD133-positive cells exceeded 1% of the total. Then, the hUCBMNCs were collected and washed twice in DMEM. The final cell-based product was cryopreserved by 2 ml sterile cryovials with 10% DMSO, and the desired cell concentration should reach 3 × 10^7^ MNCs/ml. The hUCBMNCs used in the animal experiments were examined by the China National Institutes for Food and Drug Control (NIFDC). For purpose of excluding the influence of individual differences, the human umbilical cord blood sample was from the same full-term placenta of a healthy woman donor at the same injection time. This study protocol was approved by the Medical Ethics Committee of the Second Xiangya Hospital of Central South University. This project has received a certificate of approval from the Chinese Clinical Trial Registry (ChiCTR1800019254).

### 2.3. Animal Experiment and Cell Experiment Process

#### 2.3.1. Establishment of LPS-Induced AKI Rat Model

**Figure 1:**
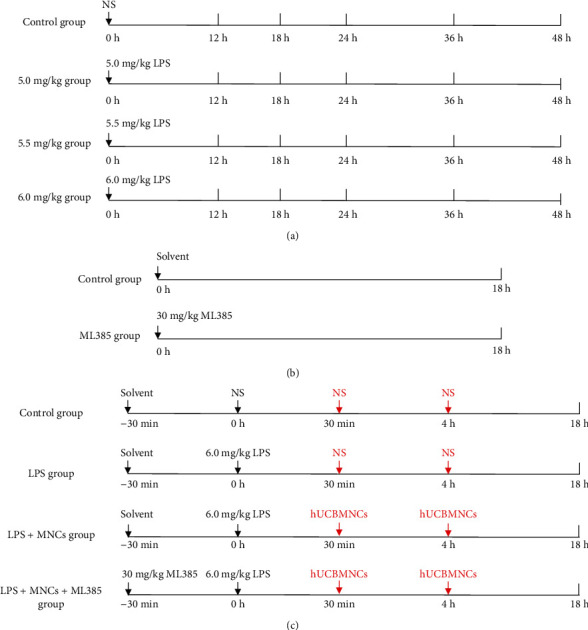
Flow diagrams of animal experiments. NS: normal saline; LPS: lipopolysaccharide; hUCBMNCs: human umbilical cord blood mononuclear cells. (a) Preliminary experiment. (b) ML385 toxicity and reliability experiment. (c) Intervention experiment.

A model for LPS-induced AKI in rats was developed in which animals were injected intravenously with LPS. Twenty male adult SD rats were selected and divided into four groups randomly (Figure 1(a)): control group (10 ml/kg NS, i.v., *n* = 5), 5.0 mg/kg group (5.0 mg/kg LPS, i.v., *n* = 5), 5.5 mg/kg group (5.5 mg/kg LPS, i.v., *n* = 5), and 6.0 mg/kg group (6.0 mg/kg LPS, i.v., *n* = 5). Blood samples were collected using capillary glass tubes from orbital venous plexus to test serum creatinine (SCr) and blood urea nitrogen (BUN) at 0 h, 12 h, 18 h, 24 h, 36 h, and 48 h. Mean arterial pressure (MAP) was recorded using a tail-cuff system, and the body temperature was measured in the anus at the same time. Animals were sacrificed at 48 h to gather kidney tissues for further experiments. Different evaluation parameters were used to evaluate the degree of inflammation, oxidative stress, and renal damage. According to these evaluation parameters, the optimal rat model of LPS-induced AKI was built.

#### 2.3.2. Explore the Acute Toxicity and Reliability of ML385

No acute toxicity of ML385 in animal experiments has been reported. In order to further explore the toxicity and reliability of ML385 in the important organs of rats, six adult male SD rats were randomly divided into the control group and the ML385 group (Figure 1(b)). The rats in the ML385 group (*n* = 3) received injections of ML385 (dissolved in DMSO, 30 mg/kg) intraperitoneally. The rats in the control group (*n* = 3) received the same volume injections of 5% DMSO intraperitoneally. Blood samples were collected from orbital venous plexus before the injection to test SCr, BUN, blood glucose (GLU), lactic dehydrogenase (LDH), alanine aminotransferase (ALT), and aspartate aminotransferase (AST). All animals were sacrificed at 18 h, and the samples of the hearts, livers, spleens, lungs, and kidneys were harvested for further experiments.

#### 2.3.3. The Efficacies of hUCBMNCs on LPS-Induced AKI and Related Mechanism Study in Rats

The cells were evaluated for viability before cell transplantation, using trypan blue. Finally, the cells were diluted and the final injection concentration was 2 × 10^6^ MNCs/ml [16]. Thirty-two adult male SD rats were divided into four groups randomly (8 rats in each group): the control group, LPS group, LPS+MNCs group, and LPS+MNCs+ML385 group (Figure 1(c)). ML385 was injected intraperitoneally 30 minutes before LPS injection. At *t* = 0 h, all rats received an equal volume of NS or LPS 6 mg/kg intravenously. At the points of 30 minutes and 4 hours after LPS injection, animals were injected with 2 × 10^6^ hUCBMNCs through the tail vein. Animals were killed at *t* = 18 h, the point of time for optimum effect.

#### 2.3.4. Cell Experiment Protocol

In order to establish an LPS-induced cell injury model, human tubular epithelial (HK-2) cells were treated with 10, 20, or 30 *μ*g/ml LPS for 6 h to select an optimal dosage of LPS. Then, HK-2 cells were treated with the optimal dosage of LPS for 0 h, 4 h, 6 h, or 8 h. According to the expression of Nrf2, HO-1, p62, LC3-II, and cleaved caspase 3, the optimal model of LPS-induced cell injury in HK-2 cells was established.

To elaborate the protective mechanism of the Nrf2 pathway, tert-butylhydroquinone (TBHQ, Selleckchem, Houston, TX, USA; Cat# S4990) and 3-methyladenine (3-MA, Selleckchem, Houston, TX, USA; Cat# S2767) were used to activate Nrf2 and inhibit autophagy, respectively. HK-2 cells were divided into four groups: the control group, LPS group, LPS+TBHQ group, and LPS+TBHQ+3-MA group, which were treated with LPS or LPS plus TBHQ 40 ng/ml or (and) 3-MA 10 mM for indicated times.

### 2.4. Hematoxylin and Eosin Staining

4% paraformaldehyde was used to fix the kidneys, hearts, livers, spleens, and lungs. Then, the samples were embedded in paraffin and then used to prepare 3 *μ*m thick sections [17]. The sections were then stained with hematoxylin and eosin staining. Ten high-magnification (×200) fields of the cortex and outer stripe of the outer medulla were selected by two renal pathologists who were experienced and blinded to these animal experiments. Kidney tubular damage was scored as follow-up for the semiquantitative analysis [18]: no injury (0), <25% (1), 25-50% (2), 50-75% (3), and >75% (4).

### 2.5. Ultrastructural Examination

Kidney or cell samples were first fixed with 2.5% glutaraldehyde, and then, procedures including conventional dehydration, osmosis, embedding, sectioning, and staining were conducted, as previously described [17]. Finally, the ultrastructure of cells was observed under a TEM.

### 2.6. Terminal Deoxynucleotidyl Transferase dUTP Conjugated with Fluorescein (TUNEL) Assay

Renal tissues of rats were gathered and apoptotic cells were evaluated by a DNA Fragmentation Imaging Kit (In Situ Cell Death Detection kit; Roche, Basel, Switzerland). Ten random fields were selected under a fluorescence microscope (Motic, BA410E) to calculate the average number of TUNEL-positive cells in each group.

### 2.7. Determination of Blood Parameters

Blood cell counting and classification were determined by an automatic blood cell analyzer in the clinical laboratory of the Second Xiangya Hospital of Central South University. SCr, BUN, ALT, AST, GLU, and LDH were detected using the automatic biochemical analyzer in the laboratory department of Second Xiangya Hospital of Central South University (Hitachi 7170A, Japan). The rat C Reactive Protein ELISA Kit (CSB-E07922r, Hcusabio, Wuhan, China) was used to test the C reactive protein (CRP) according to the directions.

### 2.8. Measurement of Malondialdehyde (MDA), Superoxide Dismutase (SOD) Oxidase Activities, and Reactive Oxygen Species (ROS) in Serums or Renal Tissues

Commercial kits (Jiancheng Bioengineering Institute, Nanjing, China) were used to test MDA concentration (TBA method, A003-1) and SOD activity (WST-1 method, A001-3) with the manufacturer's instructions.

Dihydroethidium (DHE, Thermo Fisher Scientific, D11347) staining was adopted to measure ROS in renal tissues according to related articles [19, 20]. To put it simply, the whole process could be divided into three parts. First, frozen sections of renal tissues were prepared after the rat kidneys were harvested. Slides containing cryostat sections of the kidneys could be stored at −80°C. And then, the cryosections were incubated with 10 *μ*M DHE for 30 min in a dark and humidified room at room temperature and then counterstained with diamidine phenyl indole (DAPI, Sigma-Aldrich, D9542). Finally, fluorescent photos were taken with a fluorescence microscope and fluorescence intensity was determined semiquantitatively by Image J software.

### 2.9. Western Blotting

Renal tissues and cells were lysed to obtain the total proteins, quantifying with a BCA kit for concentration [17]. Then, protein samples were separated by SDS-polyacrylamide gels with suitable concentration and then transferred to PVDF membranes, and the membranes were blocked with 5% bull serum albumin and immunoblotted with primary antibodies overnight and subsequently incubated by a horseradish peroxidase-conjugated antibody. Primary antibodies NLRP3 (ab214185, Abcam), Keap1 (60027-1-Ig, Proteintech), Nrf2 (16396-1-AP, Proteintech), Drp1 (ab184247, Abcam), mfn2 (12186-1-AP, Proteintech), PINK1 (ab23707, Abcam) Parkin (ab179812, Abcam), p62 (ab109012, Abcam), *β*-actin (20536-1-AP, Proteintech), *β*-tubulin (GB11017, Servicebio, Wuhan), GAPDH (10494-1-AP, Proteintech), HMGB1 (DF7008,Affinity Biosciences), HO-1 (27282-1-AP, Proteintech), cleaved caspase 3 (9664S, Cell Signal Technology), and LC3-II (3868S, Cell Signal Technology) were used following the manufacturer's recommended dilutions. Image J software was used for semiquantitative calculation.

### 2.10. Statistical Analysis

The statistical software SPSS version 18.0 was used to perform a statistical analysis. A paired *t*-test was performed for comparison between two groups. One-way ANOVA was performed for multiple group comparisons. All data were presented as mean values ± standard error (*mean* ± *SE*). The level of statistical significance was set at *p* < 0.05.

## 3. Results

### 3.1. Establishment of LPS-Induced AKI Model

After the injection of LPS, all rats developed lethargy, ocular and nasal bleeding, and diarrhea in varying degrees. Markedly, after 6.0 mg/kg LPS injection, two rats died within 12 to 24 h and no rat in other groups died. In rats with 6.0 mg/kg LPS injection, the highest SCr was achieved at 18 h, which met the diagnostic criteria for AKI in KDIGO. Other hematologic indicators also suggested that the inflammatory response, oxidative stress, and kidney damage were the most severe in the 6.0 mg/kg group. In rats injected with 6.0 mg/kg LPS, renal histological examination revealed more extensive necrosis of renal tubular epithelial cells and loss of brush border, and mesangial cell proliferation could be observed. More details could be seen in Supplemental Fig. 1 and Supplemental Table 1. Based on the above results, we thought it was suitable to select the dosage of 6.0 mg/kg LPS and 18 h after injection to build the rat model of LPS-induced AKI.

### 3.2. The Safety and Reliability of ML385 Injection in the Short Term of Rats

**Figure 2:**
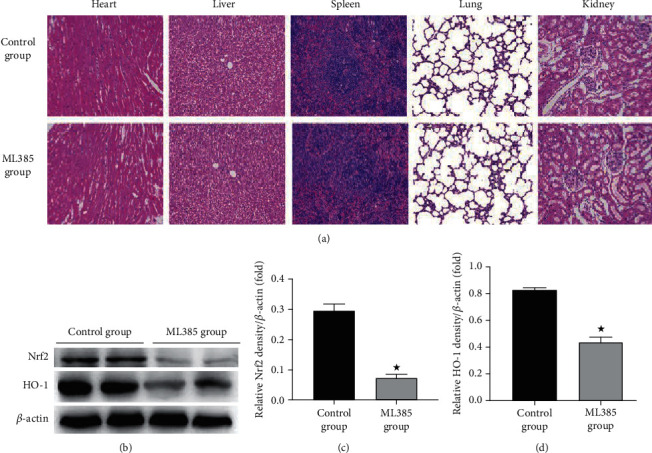
The acute toxicity and reliability of ML385. We observed morphological changes of the heart, liver, spleen, lung, and kidney of treated rats under ML385. Renal tissues were collected for Western blot analysis. (a) Representative photomicrographs of the heart, liver, spleen, lung, and kidney in rat with and without ML385 treatment (original magnifications: ×200; hematoxylin and eosin stain). (b) Representative blots of Nrf2, HO-1, and *β*-actin. (c) Densitometry of Nrf2. (d) Densitometry of HO-1. Values are means ± SE. ^★^*p* < 0.05 versus the control group.

According to the results in Table 1, no significant change could be seen in serological indexes which could reflect the function of viscera and body weight at the same time point in each group. There was no difference in hematologic parameters except GLU between the control group and ML385 group. Because the experiment started at 4 pm and ended at 10 am the next day, the increase of GLU at 18 h could be explained by feeding. The levels of MDA and SOD in the kidney tissue and serum were the same in these two groups (Table 2). As shown in Figure 2(a), there was no significant change in the histopathology of the kidney, liver, heart, spleen, and lung after the injection of ML385 compared with the control group. Hence, these results proved the safety of the ML385 injection in the short term.

Left renal tissues of rats in each group were collected for immunoblot analysis. HO-1 is a downstream effector molecule of Nrf2 which has antioxidant effects. The expression levels of Nrf2 and HO-1 were significantly declined in the ML385 group (Figures 2(b)–2(d)), indicating the reliability of ML385.

### 3.3. Inhibition of Nrf2 Activity Partly Weakened Protection of hUCBMNCs in LPS-Induced Renal Injury and Inflammation in Rats

**Figure 3:**
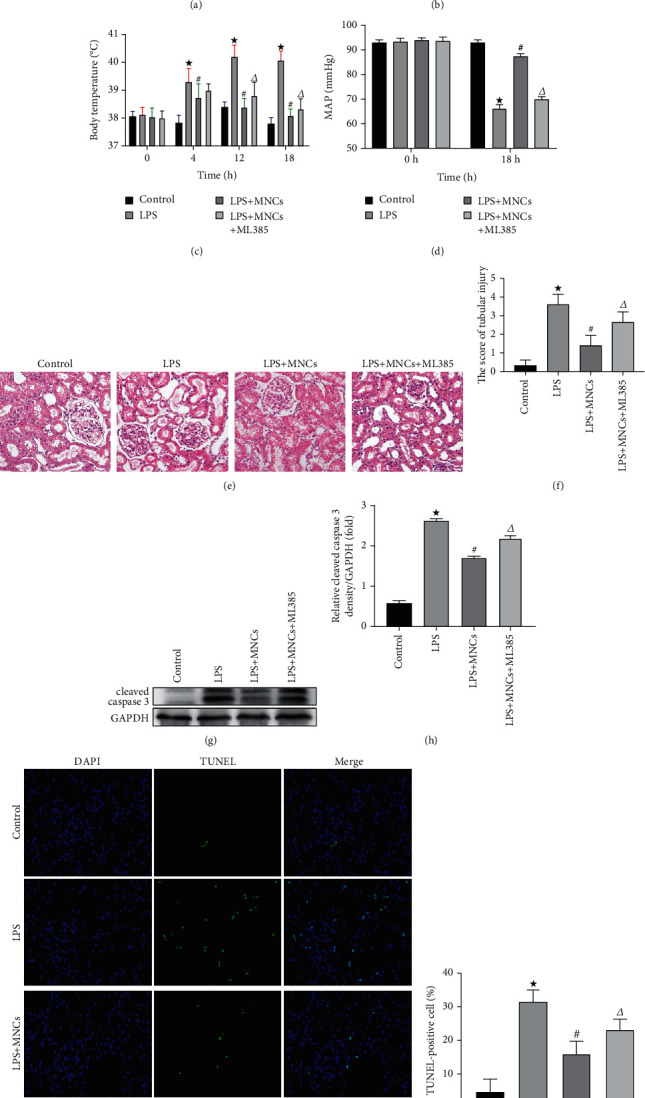
The changes of blood parameters and renal damages in intervention experiment. We observed the changes of SCr, BUN, body temperature, MAP, and apoptosis of treated rats in the control group, LPS group, LPS+MNCs group, and LPS+MNCs+ML385 group. (a) Changes of SCr level in different groups. (b) Changes of BUN level in different groups. (c) Changes of body temperature level in different groups. (d) Changes of MAP level in different groups. (e) Representative photomicrographs of tubular cell injury in rat kidney tissue sections of the control group, LPS group, LPS+MNCs group, and LPS+MNCs+ML385 group (original magnifications: ×200; hematoxylin and eosin stain). (f) Semiquantitative analysis of histologic scoring. (g) Representative blots of cleaved caspase 3 and GAPDH. (h) Densitometry of cleaved caspase 3. (i) Representative pictures of TUNNEL assay (×200). Rat kidney tissue was stained for TUNEL (green). Nuclei were stained with DAPI (blue). (j) Apoptosis percentage. Greater than 200 cells in each group were evaluated to determine the percentage of TUNEL-positive cells. Values are means ± SE. ^★^*p* < 0.05 versus the control group. ^#^*p* < 0.05 versus the LPS group. ^∆^*p* < 0.05 versus the LPS+MNCs group.

The increase of SCr and BUN was evident in the LPS group compared to the controls (Figures 3(a) and 3(b)). In contrast, in the LPS+MNCs group, there were notable improvements in SCr and BUN, which indicated the renoprotective effects of hUCBMNCs. But interestingly, we observed a deterioration of SCr and BUN in the LPS+MNCs+ML385 rats compared to the LPS+MNCs group. Hematoxylin and eosin staining was used to observe the changes of renal histomorphology, and the tubular pathological scores were calculated (Figures 3(e) and 3(f)). The control group was almost normal. Severe tubular epithelial cell vacuolization and shedding, brush border rarefaction, and tubular dilation were observed in the LPS group. Less tubular dilation, vacuolization, and brush border rarefaction were detected in the LPS+MNCs group. The histopathological damage in the LPS+MNCs+ML385 group was more serious than that in the LPS+MNCs group but less severe than that in the LPS group. The differences between the four groups were remarkable. Furthermore, we assessed the number of apoptotic cells and the expression level of cleaved caspase 3 in renal tissues (Figures 3(g)–3(j)). The level of apoptosis in LPS rats was the highest, compared with other groups. Remarkably, hUCBMNC treatment could ameliorate apoptosis. Nevertheless, rats treated with LPS+MNCs+ML385 had a reduction in these changes when compared to the LPS+MNCs rats. These results indicated the specific role of Nrf2 in the renoprotective effects of hUCBMNCs.

**Figure 4:**
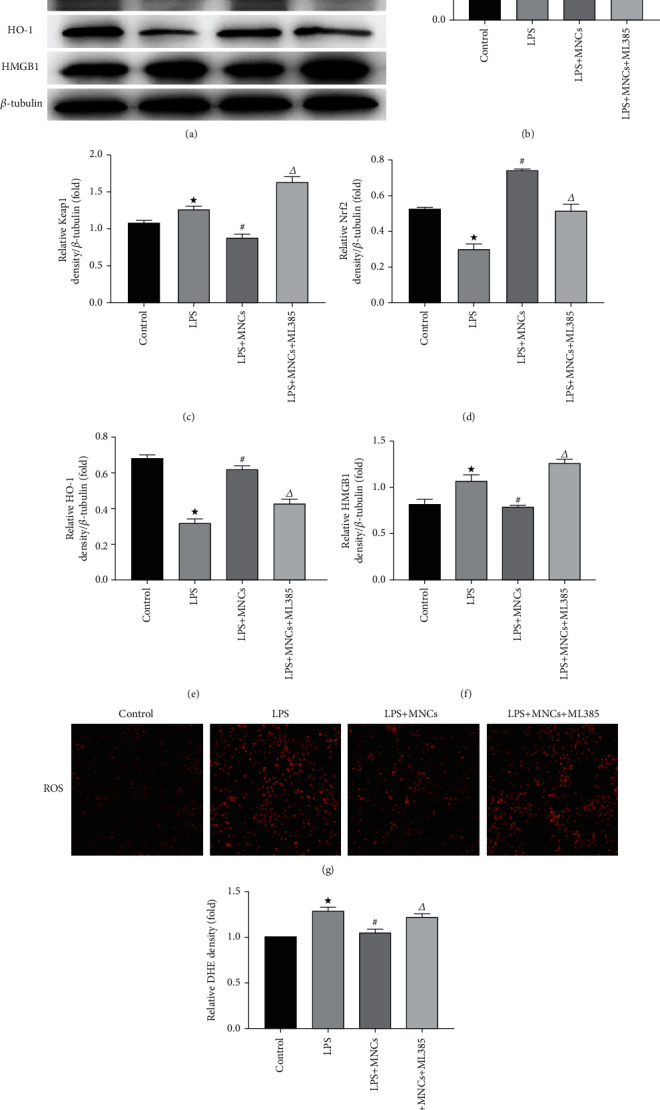
The changes of ROS, NLRP3, Keap1, Nrf2, HO-1, and HMGB1 in intervention experiment. Western blot was used to detect the expression of NLRP3, Keap1, Nrf2, HO-1, and HMGB1. Oxidative stress in kidney tubular cells was assessed using dihydroethidium (DHE). (a) Representative blots of NLRP3, Keap1, Nrf2, HO-1, HMGB1, and *β*-tubulin. (b) Densitometry of NLRP3. (c) Densitometry of Keap1. (d) Densitometry of Nrf2. (e) Densitometry of HO-1. (f) Densitometry of HMGB1. (g) Representative images of DHE staining. Original magnifications: ×200. (h) Semiquantitative analysis of DHE fluorescence intensity. Values are means ± SE, *n* = 6 for each group. ^★^*p* < 0.05 versus the control group. ^#^*p* < 0.05 versus the LPS group. ^∆^*p* < 0.05 versus the LPS+MNCs group.

As shown in Table 3, HGB and PLT of the LPS group were significantly decreased and, WBC, neutrophils, and serum CRP were significantly increased compared with controls. And the body temperature increased and the MAP decreased in the LPS group when compared to the controls (Figures 3(c) and 3(d)). NLRP3 and HMGB1 act as indicators of the severity of inflammation reaction. Western blot assay showed that the expression of NLRP3 and HMGB1 in renal tissues increased after LPS injection (Figures 4(a), 4(b), and 4(f)). These data indicated that severe infection and shock occurred in rats after LPS injection. In contrast, in the LPS+MNCs group, there were some marked improvements in body temperature, MAP, and hematologic parameters, indicating an anti-inflammatory effect of hUCBMNCs. The hUCBMNCs also had an obvious effect on decreasing the expression of NLRP3 and HMGB1 in the kidney. However, a deterioration of inflammation reaction parameters was detected in LPS+MNCs+ML385 rats. Hence, we confirmed that the protective effects on LPS-induced severe renal injury and inflammatory reaction of hUCBMNCs were mediated by Nrf2.

### 3.4. hUCBMNCs Reduced the Level of Oxidative Stress in the Rat Model

We measured the level of SOD and MDA, both circulating and renal tissue level (Table 3). There was no significant difference in MDA in renal tissues among these four groups. MDA in the serum was remarkably increased, and SOD in tissues was significantly decreased in the LPS group and LPS+MNCs+ML385 group (*p* < 0.05). The treatment of hUCBMNCs could ameliorate these changes. The level of SOD in the serum was reduced in the other three groups in different degrees compared to the controls. However, the LPS+MNCs group had the smallest reduction (*p* < 0.05). ROS level in renal tissues was examined by DHE staining (Figures 4(g) and 4(h)). A significant increase of ROS was exhibited in the kidneys of rats with LPS injection, and animals treated with hUCBMNCs had a reduction in the generation of ROS in kidney tissues. Compared to rats treated by LPS+MNCs, rats with LPS+MNCs+ML385 treatment had a higher level of ROS. Thus, ML385 could partly reduce these effects when compared to the LPS+MNCs group.

We measured the expression levels of Keap1, Nrf2, and HO-1 in the kidneys by Western blot (Figures 4(a) and 4(c)–4(e)). Keap1-Nrf2 is a classical antioxidant regulatory pathway in the organism. Rats from the LPS group had significantly lower Nrf2 and HO-1 and higher Keap1 compared to the control group. The rats in the LPS+MNCs group had a significantly higher level of Nrf2 activity compared to the LPS rats, which suggested that hUCBMNC treatment could prevent the decreasing of the Nrf2 activity seen in LPS rats. However, the antioxidant role of hUCBMNCs was partly abolished by inhibiting Nrf2. These results indicated that the hUCBMNCs could protect against LPS-induced AKI via the Nrf2 pathway.

### 3.5. hUCBMNCs Improved Mitochondrial Damage and Autophagy in the LPS-Induced AKI via the Nrf2 Pathway in Rats

**Figure 5:**
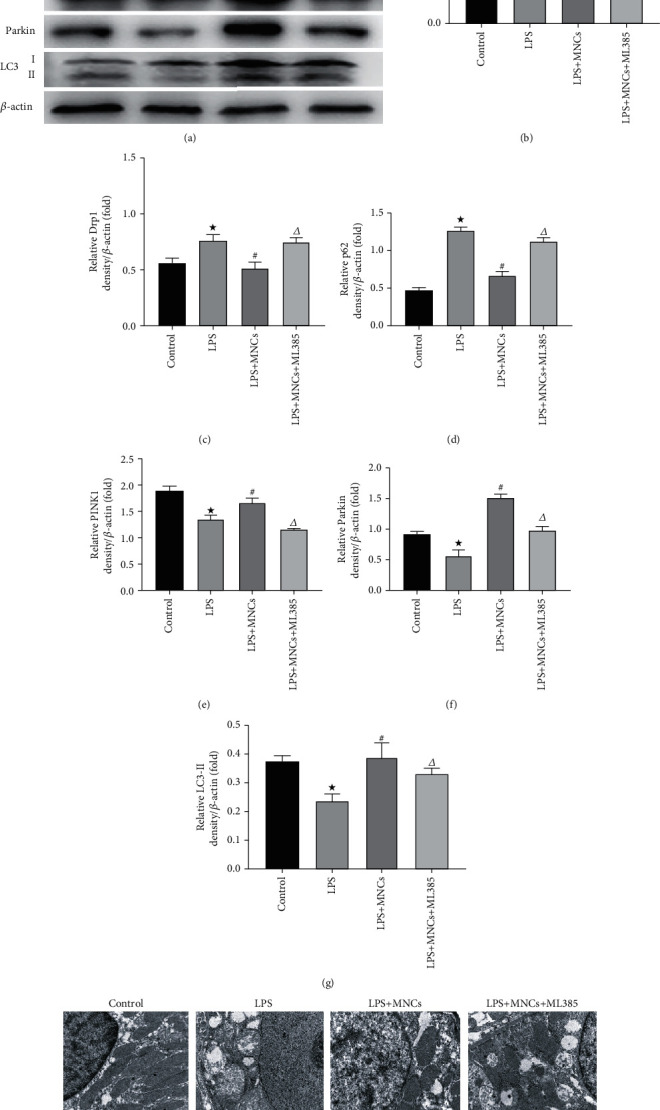
The changes of mitochondrial damage and autophagy-associated proteins in intervention experiment. Western blot was used to detect the expression of mfn2, Drp1, p62, PINK1, Parkin, and LC3. Mitochondria injury in rat kidney tissues was observed by transmission electron microscopy (TEM). (a) Representative blots of mfn2, Drp1, p62, PINK1, Parkin, LC3, and *β*-actin. (b) Densitometry of mfn2. (c) Densitometry of Drp1. (d) Densitometry of p62. (e) Densitometry of PINK1. (f) Densitometry of Parkin. (g) Densitometry of LC3-II. (h) Representative photomicrographs of mitochondria injury in rat kidney tissues (original magnifications: ×10000, under a Hitachi H7700 electron microscope). Values are means ± SE, *n* = 6 for each group. ^★^*p* < 0.05 versus the control group. ^#^*p* < 0.05 versus the LPS group. ^∆^*p* < 0.05 versus the LPS+MNCs group.

In order to further confirm the relevant mechanism of the renoprotective effects of hUCBMNCs, we observed the changes of the mitochondria and autophagy-related protein expression in the kidneys. Profission proteins (Drp1) and profusion proteins (mfn2) participate in maintaining the mitochondrial network balance. PINK1 and Parkin are indicators of mitochondrial damage [21]. As shown in Figures 5(a)–5(g), LPS induced the decreased expression of mfn2, PINK1, Parkin, and LC3-II and increased expression of Drp1 and p62. These changes could be reversed by hUCBMNC treatment. Interestingly, these effects of hUCBMNCs were partly abolished by ML385 treatment. TEM was adopted to observe changes in the mitochondria (Figure 5(h)). Obvious break and disappearance of the mitochondrial cristae, turgidity, disarrangement, and vacuolization of the mitochondria could be seen in the rats injected with LPS. Compared to the LPS group, less noticeable disarrangement and vacuolization of the mitochondria were observed in the LPS+MNCs group. The changes of the mitochondria in the LPS+MNCs+ML385 group were similar with rats injected with LPS. Taken together, hUCBMNCs could improve mitochondrial damage and increase autophagy-related protein expression in the LPS-induced AKI rat model via the Nrf2 pathway.

### 3.6. The Establishment of LPS-Induced Cell Injury Model

We further explored the establishment of LPS-induced cell injury model. In the in vitro culture, HK-2 cells were treated with LPS at different concentrations and durations (Supplemental Fig. 2). Compared to other concentrations, cells treated with 30 *μ*g/ml LPS for 6 h had the highest level of apoptosis and the lowest level of autophagy. Meanwhile, the decline in the Nrf2 expression led to a remarkable drop in HO-1 in this group. According to the above experimental results, 30 *μ*g/ml LPS and culture duration of 6 h were selected for the following cell experiments. These results also indicated that LPS could significantly downregulate the Nrf2 signaling pathway and restrain autophagy in this LPS-induced cell injury model.

### 3.7. Activation of Nrf2 Pathway Reduced Inflammation, Apoptosis, and Mitochondrial Damage via Autophagy in HK-2 Cells

**Figure 6:**
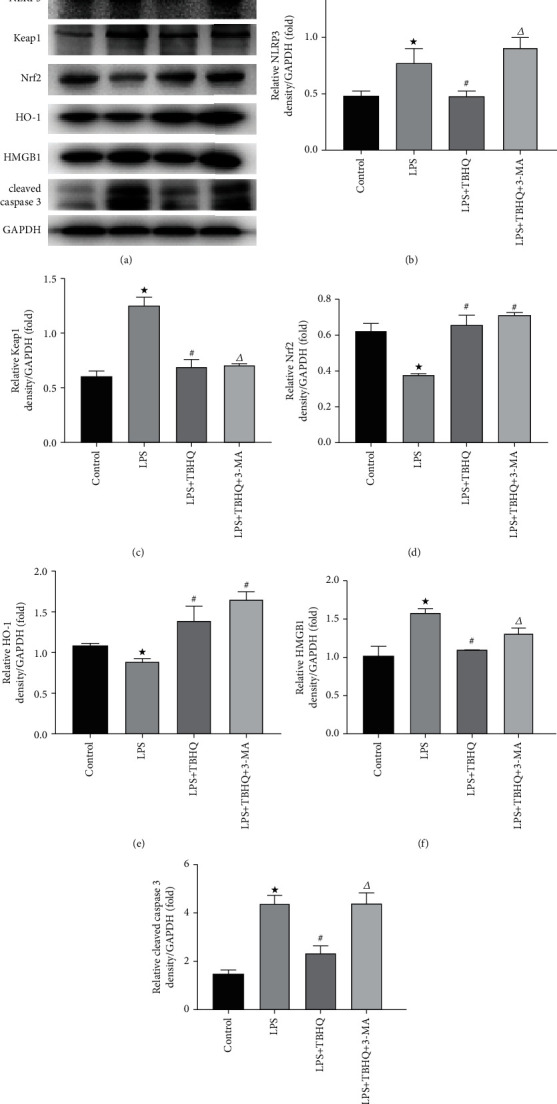
The changes of NLRP3, Keap1, Nrf2, HO-1, HMGB1, and cleaved caspase 3 of cells. Western blot was used to detect the expression of NLRP3, Keap1, Nrf2, HO-1, HMGB1, and cleaved caspase 3 of cells in the control group, LPS group, LPS+TBHQ group, and LPS+TBHQ+3-MA group. (a) Representative blots of NLRP3, Keap1, Nrf2, HO-1, HMGB1, cleaved caspase 3, and GAPDH. (b) Densitometry of NLRP3. (c) Densitometry of Keap1. (d) Densitometry of Nrf2. (e) Densitometry of HO-1. (f) Densitometry of HMGB1. (g) Densitometry of cleaved caspase 3. Values are means ± SE, *n* = 6 for each group.^★^*p* < 0.05 versus the control group. ^#^*p* < 0.05 versus the LPS group. ^∆^*p* < 0.05 versus the LPS+TBHQ group.

**Figure 7:**
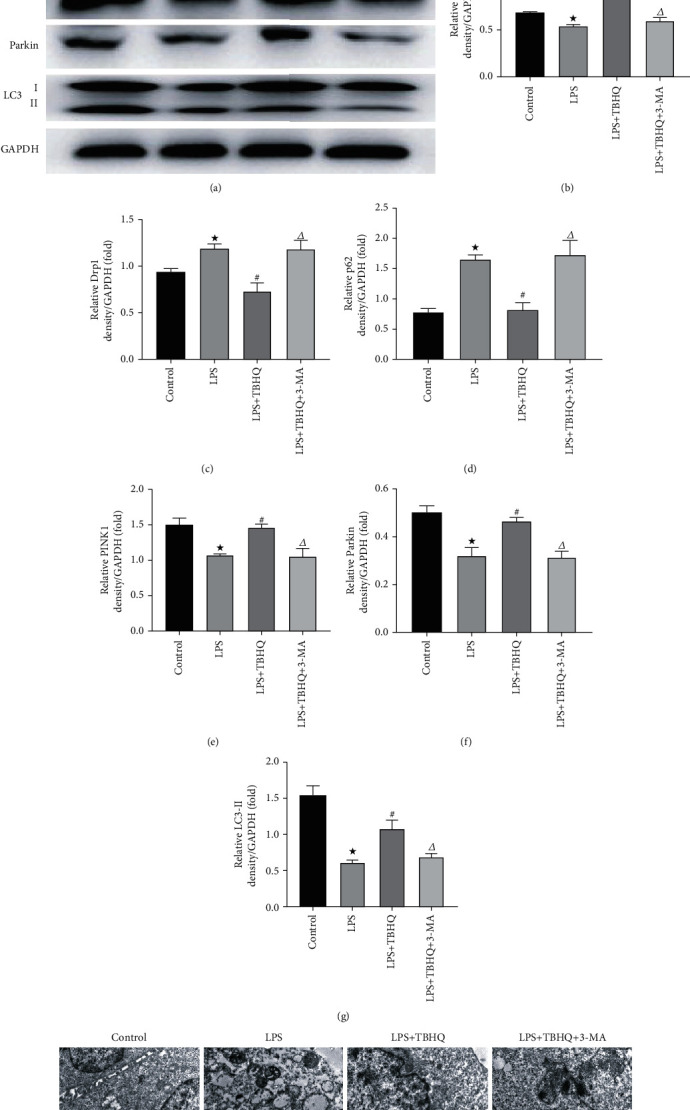
Ultrastructural changes and the changes of mitochondrial damage-associated proteins and autophagy-associated proteins of cells. Western blot was used to detect the expression of mfn2, Drp1, p62, PINK1, Parkin, and LC3-II. Mitochondria injury in rat kidney tissues was observed by transmission electron microscopy (TEM). (a) Representative blots of mfn2, Drp1, p62, PINK1, Parkin, LC3-II, and GAPDH. (b) Densitometry of mfn2. (c) Densitometry of Drp1. (d) Densitometry of p62. (e) Densitometry of PINK1. (f) Densitometry of Parkin. (g) Densitometry of LC3-II. (h) Representative photomicrographs of cells. Typical autophagosome (red arrow) was observed in the LPS+TBHQ group (original magnifications: ×10000, under a Hitachi H7700 electron microscope). Values are means ± SE, *n* = 6 for each group. ^★^*p* < 0.05 versus the control group. ^#^*p* < 0.05 versus the LPS group. ^∆^*p* < 0.05 versus the LPS+TBHQ group.

The expression levels of proteins that were associated with inflammation, apoptosis, and mitochondrial damage were detected by immunoblotting (Figures 6 and 7). Compared to the control group, NLRP3 and HMGB1 expressions were increased in HK-2 cells with LPS treatment. Apoptosis level was also increased in HK-2 cells treated by LPS, evidenced by cleaved caspase 3 increasing. Cells from the LPS group had significantly lower Nrf2 and HO-1 and higher Keap1 compared to the control. As for autophagy and mitochondrial damage, LPS induced the decreased expression of mfn2, PINK1, Parkin, and LC3-II and increased expression of Drp1 and p62. As shown in Figure 6, TBHQ could activate the Nrf2 pathway effectively and 3-MA could reduce the autophagy level significantly. The activation of Nrf2 could remarkably reduce inflammation, apoptosis, and increase autophagy, which was demonstrated by the decrease of NLRP3, HMGB1, cleaved caspase 3, Drp1, and p62 and increase of mfn2, PINK1, Parkin, and LC3-II. However, these effects of TBHQ were partly abolished by 3-MA treatment. Mitochondria damage, autophagy, and apoptosis were also observed by TEM (Figure 7(h)). Clear nucleolus and continuous karyotheca were shown in the control group. The mitochondrial morphology was normal and the mitochondria were clear. LPS treatment induced mitochondrial swelling, mitochondrial pyknosis, and mitochondrial fragmentation. Other symptoms observed which represented apoptosis included the following: the karyotheca dissolved, the nucleolus disappeared, and the nuclear chromatin gathered. In cells with LPS+TBHQ treatment, most mitochondria were clear. The nucleolus of the cell was clear, and karyotheca was continuous. Typical autophagosomes could be observed. Nucleus pyknosis, chromatin aggregation, mitochondrial malformation, pyknosis, swelling, and dissolution could be seen in the LPS+TBHQ+3-MA group. The injuries in the LPS+TBHQ+3-MA group were remarkably worse than those in the LPS+TBHQ group. Therefore, we demonstrated that activating the Nrf2 pathway could play a protective role in LPS-induced AKI by regulating autophagy at the cellular level.

## 4. Discussion

One of the salient findings of our study is that we first substantiated that hUCBMNCs could reduce renal damage in the LPS-induced AKI via increasing autophagy and attenuating oxidative stress, inflammation, and mitochondrial damage. We detected that hUCBMNCs protected against LPS-induced AKI in rats, at least in part, via the Nrf2 pathway. The cell experiments were further conducted to elaborate the protective mechanism of the Nrf2 pathway. The results showed that activation of the Nrf2 pathway could protect HK-2 cells from LPS-induced injury through increasing autophagy and decreasing mitochondria injury.

Sepsis and septic shock bring serious economic burden to health management. AKI can complicate sepsis or septic shock frequently, which is associated with longer hospital stay and an increased risk for death independently [22, 23]. The minimum quality threshold in preclinical sepsis studies (MQTiPSS) summarizes the present studies and provides some considerations about sepsis models [24]. According to the recommendation, LPS in the study of the first 24 hours of the septic response is relevant. Former studies have tried either 10 *μ*g/kg LPS in pigs [25] or 20 mg/kg LPS in rats [26] to induce AKI animal models. Injection of 15 *μ*g/g LPS intraperitoneally two times also led to AKI in mice [27]. However, the majority of studies did not mention reasons for dose selection and experimental details reflected organ dysfunction. The mode of administration, the number of injections, and dosage of LPS intervention vary widely, which may be related to LPS serotypes, animal species, cell types, and target protein expression level. In our study, we explored the curve of SCr and BUN with time under different LPS dosage. In addition to evaluating the kidney function of rats, we also examined the indicators which could reflect the severity of sepsis. If the detailed information of our animal model is fully utilized and discussed, it will provide more basic data for future research. The existing research about the LPS concentrations in cell experiments ranges from 50 ng/ml to 10 *μ*g/ml [28, 29] and the duration ranges from 30 minutes to 48 hours [29, 30]. According to our experimental results, 30 *μ*g/ml LPS and culture duration of 6 h were selected for the cell model. Prior to our research, there are no relevant reports on the LPS serotype 0111: B4 intervention in the HK-2 cells. Hence, we believe our data about the establishment of in vivo and in vitro models are valuable.

AKI is one of the common complications of sepsis, mainly manifested as renal tubular injury [31]. Heretofore, there is no effective treatment for septic AKI. In recent years, many studies have shown that stem cells are effective in tissue regeneration and repair and have broad prospects in the treatment of kidney diseases. Much of the research in cell therapy has focused on nephropathy, such as endothelial progenitor cells and mesenchymal stem cells [32, 33]. As a valuable source of cells, hUCB is increasingly acquainted for therapeutic use [34]. hUCBMNCs which are cells with one nucleus in umbilical cord blood, are isolated and purified by density gradient centrifugation. hUCBMNCs are a hybrid system containing immature immune cells, HSCs, MSCs, EPCs, and so forth. Recent studies have revealed that besides treating hematological malignancies, hUCBMNCs show good therapeutic effects on cerebral ischemia, chronic complete spinal cord injury, erectile dysfunction, and ischemic injury of ventricular [9, 11–13]. In the research of nephrology, hUCBMNCs can reduce SCr and BUN in the rats with diabetic nephropathy [10]. hUCBMNCs also can improve the renal function of nephrotoxic kidney [35]. In the studies above, there was no standard for the dosage of hUCBMNCs. The dosage of hUCBMNC injection in an animal experiment ranges from 0.2 × 10^6^ to 30 × 10^6^. In our previous study, multiple injections of hUCBMNCs (2 × 10^6^ hUCBMNCs a time) have a great benefit to the treatment for renal interstitial fibrosis in cisplatin-treated rats [36]. However, it is unclear whether hUCBMNCs get a satisfactory effect on protecting septic AKI. In this study, we found that hUCBMNCs could alleviate renal damage in rats with LPS injection. Notably, the level of inflammation, oxidative stress, and apoptosis was attenuated by hUCBMNCs treatment. As we know, it was the first time to elucidate the ability of hUCBMNCs in defending against LPS-induced AKI in an animal model study.

Although sepsis is one of the main causes of AKI, the potential mechanisms of septic AKI are not completely understood. The underlying mechanisms may contain endothelial dysfunction, infiltration of inflammatory cells, hemodynamic changes, thrombosis, and obstruction of tubules with necrotic cells and debris [37–40]. These situations ultimately magnify the signal of inflammation and cause more serious oxidative stress. Oxidative stress is an important part in the pathophysiological mechanism of the AKI after sepsis. Animal experiments have shown that reactive nitrogen species and ROS are involved in renal tubular epithelial cell injury in sepsis [41]. Generally, Keap1-Nrf2/ARE is a classical antioxidant regulatory pathway in the organism. Nrf2 normally binds with Keap1 remaining suppressed. However, the degradation of Nrf2 is inhibited under stress and nuclear translocation occurs. In the kidneys of the rat model, LPS insult has been shown to increase ROS, MDA, and the level of Keap1 protein and inhibit SOD and the expression of the Nrf2/HO-1 pathway. Our findings indicated that hUCBMNCs had an obvious effect on reducing oxidative stress when compared to the LPS group. We also found that the Nrf2 and HO-1 expression levels of rats in the LPS+MNCs group were significantly higher than that of rats in the LPS group. ML385 is a specific Nrf2 inhibitor which can affect the expression of downstream genes in the Nrf2 pathway [42]. Interestingly, rats with the LPS+MNCs+ML385 treatment had a higher level of oxidative stress compared to rats treated by LPS+MNCs. These results indicated that ML385 could partly reduce the protective effects of hUCBMNCs and also proved that the renoprotective effect of the hUCBMNCs was related to the Nrf2 pathway.

Autophagy is an evolutionarily conserved, dynamic process of degradation of intracellular organelles and macromolecules by lysosomal hydrolases [7]. Previous studies suggest that the low level of autophagy is shown when AKI is detected in septic AKI models [43, 44]. After activating, Nrf2 can bind with the antioxidant response element and regulate downstream genes under stress, including antioxidant and autophagy genes, such as HO-1 and p62. There are many studies about the relationship between the Nrf2 pathway and autophagy in the ischemia-reperfusion AKI, toxic AKI, and diabetic nephropathy [7]. HO-1-induced autophagy can provide protective effects of podocytes with high-glucose treatment [45]. However, the relationship between Nrf2 and autophagy in septic AKI has not been clearly studied. We demonstrated the expression levels of Nrf2, HO-1, and LC3-II could be increased and p62 could be decreased by hUCBMNCs in LPS-induced AKI rat model. We further designed cell experiments to elaborate the protective mechanism of Nrf2. Activating Nrf2 could attenuate apoptosis and mitochondrial damage significantly in HK-2 cells with LPS treatment. Typical autophagosomes could be observed in cells with an Nrf2 activator. After inhibiting autophagy with 3-MA, the protective effect of Nrf2 was weakened. So, it was proved that Nrf2 could play an important role in cell protection by improving the level of autophagy.

Although we have illuminated these innovative findings of our study above, the limitations of this study would be clarified. First, differences do exist between LPS exposure and clinical situations. Therefore, limitations may be unavoidable in extrapolating the conclusions to clinical sepsis. Further studies about hUCBMNC renoprotective effects are needed in other sepsis model. Second, the hUCBMNCs used in this experiment are composed of a mixture of immature immune cells and multiple stem cell types. The stem cell population of the hUCBMNCs is very small, the majority of the cells being immune cells. The effectiveness of hUCBMNC treatment may be related to the regulation of immune response, automatic homing of stem cells, secretion, and humoral effects of cytokines [46]. Further studies are needed to explore the role of the specific components in the effect of renal protection. Finally, gene knockout animal and cell models are needed for further research on related molecular mechanisms.

## 5. Conclusion

In summary, treatment with hUCBMNCs can protect against LPS-induced AKI by attenuating inflammation, oxidative stress, apoptosis, and increasing autophagy in kidney. And most importantly, this study provides evidence for the first time that the mechanism of protection of hUCBMNCs is related to the Nrf2 pathway. Activating the Nrf2 pathway can upregulate autophagy to protect LPS-induced HK-2 cell injury. The work provides enough experimental data which support the cell therapy of acute kidney injury.

## Figures and Tables

**Table 1:** Effect of ML385 injection on body weight and serum parameters in normal rats.

Parameters	Control group (*n* = 3)		ML385 group (*n* = 3)	
	0 h	18 h	0 h	18 h
Weight (g)	231.7 ± 2.5	241.7 ± 1.5^∗^	231.0 ± 2.6	241.3 ± 2.1^∆^
SCr (*μ*mol/l)	16.96 ± 1.79	18.10 ± 2.64	16.99 ± 1.43	18.27 ± 1.53
BUN (mmol/l)	4.02 ± 0.53	4.66 ± 0.85	4.17 ± 0.76	4.36 ± 0.49
ALT (U/l)	40.76 ± 4.33	43.54 ± 4.77	40.55 ± 4.27	42.31 ± 2.05
AST (U/l)	142.18 ± 4.91	146.07 ± 4.54	142.11 ± 7.05	146.97 ± 3.31
LDH (U/l)	1579.65 ± 55.78	1576.13 ± 74.82	1588.66 ± 74.86	1589.26 ± 56.57
GLU (mmol/l)	5.01 ± 0.32	11.13 ± 0.76^∗^	4.97 ± 0.40	11.64 ± 0.54^∆^

Control group: rats with injections of solvent. ML385 group: rats with injections of ML385. SCr: serum creatinine; BUN: blood urea nitrogen; ALT: alanine aminotransferase; AST: aspartate aminotransferase; LDH: lactate dehydrogenase; GLU: blood glucose. Values are means ± SE. ^∗^*p* < 0.05 versus the control group at *t* = 0 h. ^∆^*p* < 0.05 versus ML385 group at *t* = 0 h.

**Table 2:** Effect of ML385 on blood and renal tissues parameters in normal rats.

Parameters	Control group (*n* = 3)	ML385 group (*n* = 3)
RBC (10^12^/l)	5.94 ± 0.38	6.18 ± 0.17
HGB (g/l)	126.33 ± 4.16	129.00 ± 2.65
WBC (10^9^/l)	5.29 ± 0.36	5.25 ± 0.19
Neutrophil (10^9^/l)	2.20 ± 0.20	2.05 ± 0.92
PLT (10^9^/l)	958.33 ± 104.58	1024.33 ± 60.54
MDA in renal tissues (nmol/mg protein)	3.99 ± 0.25	3.97 ± 0.30
MDA in serum (nmol/ml)	4.42 ± 0.16	4.41 ± 0.23
SOD in renal tissues (U/mg protein)	14.76 ± 0.63	14.82 ± 0.56
SOD in serum (U/ml)	258.36 ± 4.24	252.43 ± 6.58

Control group: rats with injections of solvent. ML385 group: rats with injections of ML385. RBC: red blood cell; HGB: hemoglobin; WBC: white blood cell; PLT: platelet; MDA: malondialdehyde; SOD: superoxide dismutase. Values are means ± SE.

**Table 3:** Effect of hUCBMNCs on blood and renal tissue parameters in LPS-induced AKI rat model.

Parameters	Control group (*n* = 8)	LPS group (*n* = 5)	LPS+MNCs group (*n* = 7)	LPS+MNCs+ML385 group (*n* = 6)
RBC (10^12^/l)	5.73 ± 0.21	5.61 ± 0.37	5.47 ± 0.32	5.49 ± 0.44
HGB (g/l)	114.63 ± 6.25	102.8 ± 3.27^∗^	114.43 ± 5.74^∆^	107.33 ± 6.77^∗^^,#^
WBC (10^9^/l)	4.96 ± 0.49	7.20 ± 0.92^∗^	5.90 ± 0.86^∗^^,∆^	6.77 ± 1.07^∗^
Neutrophil (10^9^/l)	1.77 ± 0.57	3.47 ± 0.68^∗^	1.92 ± 0.28^∆^	3.15 ± 0.48^∗^^,#^
PLT (10^9^/l)	989.37 ± 64.92	104.00 ± 36.80^∗^	200.71 ± 74.91^∗^^,∆^	147.67 ± 35.90^∗^
CRP in serum (mg/l)	0.302 ± 0.168	6.677 ± 0.244^∗^	4.775 ± 0.259^∗^^,∆^	5.281 ± 0.151^∗^^,∆,#^
MDA in renal tissues (nmol/mg protein)	3.929 ± 0.284	4.023 ± 0.283	3.859 ± 0.293	3.991 ± 0.260
MDA in serum (nmol/ml)	4.189 ± 0.469	7.665 ± 0.393^∗^	4.367 ± 0.299^∆^	6.502 ± 0.609^∗^^,∆,#^
SOD in renal tissues (U/mg protein)	15.006 ± 0.648	11.996 ± 0.876^∗^	14.287 ± 0.891^∆^	12.115 ± 1.095^∗^^,#^
SOD in serum (U/ml)	255.175 ± 2.788	237.630 ± 2.305^∗^	243.716 ± 2.484^∗^^,∆^	236.470 ± 2.724^∗^^,#^

hUCBMNCs: human umbilical cord blood mononuclear cells; LPS: lipopolysaccharide; AKI: acute kidney injury; RBC: red blood cell; HGB: hemoglobin; WBC: white blood cell; PLT: platelet; CRP: C-reactive protein; MDA: malondialdehyde; SOD: superoxide dismutase. Values are means ± SE. ^∗^*p* < 0.05 versus the control group. ^∆^*p* < 0.05 versus the LPS group. ^#^*p* < 0.05 versus the LPS+MNCs group.

## Data Availability

The data used to support the findings of this study are available from the corresponding author upon request.

## Abbreviations

AKI: Acute kidney injury
ALT: Alanine aminotransferase
ARE: Antioxidant response element
AST: Aspartate aminotransferase
BUN: Blood urea nitrogen
CRP: C reactive protein
EPCs: Endothelial progenitor cells
GLU: Blood glucose
HSCs: Hematopoietic stem cells
hUCB: Human umbilical cord blood
hUCBMNCs: Human umbilical cord blood mononuclear cells
KDIGO: Kidney disease improving global outcomes
LDH: Lactic dehydrogenase
LPS: Lipopolysaccharide
MAP: Mean arterial pressure
MDA: Malondialdehyde
MNCs: Mononuclear cells
MQTiPSS: Minimum quality threshold in preclinical sepsis studies
MSCs: Mesenchymal stem cells
Nrf2: Nuclear factor E2-related factor 2
NS: Normal saline
ROS: Reactive oxygen species
SCr: Serum creatinine
SOD: Superoxide dismutase
TBHQ: tert-Butylhydroquinone
TEM: Transmission electron microscope
TUNEL: Terminal deoxynucleotidyl transferase dUTP conjugated with fluorescein.

## Supplementary Materials

Supplementary materials Supplemental Figure 1: the changes of blood parameters and histopathology in rats with different dosages of LPS. We observed the changes of the SCr, BUN, body temperature, and MAP of treated rats under different dosages of LPS. (A) Changes of SCr level in different groups. (B) Changes of BUN level in different groups. (C) Changes of body temperature level in different groups. (D) Changes of MAP level in different groups. (E) Representative photomicrographs of tubular cell injury in rat kidney tissue sections of different groups (original magnifications: ×200; hematoxylin and eosin stain). (F) Semiquantitative analysis of histologic scoring. Values are mean ± SE. ★*p* < 0.05 versus the other groups. Supplemental Figure 2. The establishment of LPS-induced cell injury model. Western blot was used to detect the expression of Nrf2, p62, HO-1, cleaved caspase 3, and LC3-II in cells treated with LPS at different concentrations and durations. (A) Representative blots of Nrf2, p62, HO-1, cleaved caspase 3, LC3-II, and GAPDH in cells treated with LPS at different concentrations. (B) Representative blots of Nrf2, p62, HO-1, cleaved caspase 3, LC3-II, and *β*-actin in cells treated with LPS at different durations. (C) Densitometry of Nrf2. (D) Densitometry of p62. (E) Densitometry of HO-1. (F) Densitometry of cleaved caspase 3. (G) Densitometry of LC3-II. (H) Densitometry of Nrf2. (I) Densitometry of p62. (J) Densitometry of HO-1. (K) Densitometry of cleaved caspase 3. (L) Densitometry of LC3-II. Values are mean ± SE, *n* = 6 for each group. ★*p* < 0.05 versus the other groups. #*p* < 0.05 versus cells with 30*μ*g/ml LPS 0 h and 4 h. Supplemental Table 1: Effect of different dosages LPS on blood parameters in rats at 48 h. LPS: lipopolysaccharide; RBC: red blood cell; HGB: hemoglobin; WBC: white blood cell; PLT: platelet; CRP: C-reactive protein; MDA: malonaldehyde; SOD: superoxide dismutase. Values are mean ± SE. ^∗^*p* < 0.05 versus the control. ^∆^*p* < 0.05 versus 5.0 mg/kg group. ^#^*p* < 0.05 versus 5.5 mg/kg group.
