# Supplementary material for: Role of Nrf2 in Lipopolysaccharide-Induced Acute Kidney Injury: Protection by Human Umbilical Cord Blood Mononuclear Cells
Source: Oxid Med Cell Longev. 2020 Jul 28;2020:6123459. doi: 10.1155/2020/6123459 (PMC7407026; doi:10.1155/2020/6123459)
Supplement: Supplementary materials — Supplemental Figure 1: the changes of blood parameters and histopathology in rats with different dosages of LPS. We observed the changes of the SCr, BUN, body temperature, and MAP of treated rats under different dosages of LPS. (A) Changes of SCr level in different groups. (B) Changes of BUN level in different groups. (C) Changes of body temperature level in different groups. (D) Changes of MAP level in different groups. (E) Representative photomicrographs of tubular cell injury in rat kidney tissue sections of different groups (original magnifications: ×200; hematoxylin and eosin stain). (F) Semiquantitative analysis of histologic scoring. Values are mean ± SE. ★p < 0.05 versus the other groups. Supplemental Figure 2. The establishment of LPS-induced cell injury model. Western blot was used to detect the expression of Nrf2, p62, HO-1, cleaved caspase 3, and LC3-II in cells treated with LPS at different concentrations and durations. (A) Representative blots of Nrf2, p62, HO-1, cleaved caspase 3, LC3-II, and GAPDH in cells treated with LPS at different concentrations. (B) Representative blots of Nrf2, p62, HO-1, cleaved caspase 3, LC3-II, and β-actin in cells treated with LPS at different durations. (C) Densitometry of Nrf2. (D) Densitometry of p62. (E) Densitometry of HO-1. (F) Densitometry of cleaved caspase 3. (G) Densitometry of LC3-II. (H) Densitometry of Nrf2. (I) Densitometry of p62. (J) Densitometry of HO-1. (K) Densitometry of cleaved caspase 3. (L) Densitometry of LC3-II. Values are mean ± SE, n = 6 for each group. ★p < 0.05 versus the other groups. #p < 0.05 versus cells with 30μg/ml LPS 0 h and 4 h. Supplemental Table 1: Effect of different dosages LPS on blood parameters in rats at 48 h. LPS: lipopolysaccharide; RBC: red blood cell; HGB: hemoglobin; WBC: white blood cell; PLT: platelet; CRP: C-reactive protein; MDA: malonaldehyde; SOD: superoxide dismutase. Values are mean ± SE. ∗p < 0.05 versus the control. ∆p < 0.05 versus 5.0 mg/kg group. #p < 0.05 ve [file 6123459.f1.zip › 6123459.f1/6123459.f3.docx]

Supplemental Table 1. Effect of different dosages LPS on blood parameters in rats at 48 h

|  |  | LPS | | |
| --- | --- | --- | --- | --- |
| Parameters | Control (n=5) | 5.0 mg/kg (n=5) | 5.5 mg/kg (n=5) | 6.0 mg/kg (n=3) |
| RBC (10^12^/L) | 5.03 ± 0.30 | 4.96 ± 0.46 | 5.08 ± 0.48 | 5.12 ± 0.25 |
| HGB (g/l) | 111.60 ± 3.13 | 106.40 ± 2.30^*^ | 107.00 ± 3.32 | 104.00 ± 6.00^*^ |
| WBC (10^9^/L) | 5.52 ± 0.36 | 6.69 ± 0.47^*^ | 6.86 ± 0.35^*^ | 7.23 ± 0.51^*^ |
| Neutrophil (10^9^/L) | 2.06 ± 0.60 | 2.77 ± 0.48 | 3.30 ± 0.52^*^ | 3.63 ± 0.83^*^ |
| PLT (10^9^/L) | 986.0 ± 15.0 | 69.8 ± 6.9^*^ | 50.6 ± 6.69^*,∆^ | 47.0 ± 11.3^*,∆^ |
| CRP in serum (mg/L) | 0.245 ± 0.112 | 1.329 ± 0.316^*^ | 1.510 ± 0.382^*^ | 2.799 ± 0.247^*,∆,#^ |
| MDA in serum (nmol/ml) | 4.485 ± 0.531 | 4.611 ± 1.005 | 4.729 ± 0.790 | 6.828 ± 0.822^*,∆,#^ |
| SOD in serum (U/ml) | 265.098 ± 4.012 | 259.949 ± 3.317^*^ | 254.747 ± 4.013^*,∆^ | 249.413 ± 3.639^*,∆^ |
